# Long-time follow up of physical activity level among older adults with rheumatoid arthritis

**DOI:** 10.1186/s11556-020-00242-w

**Published:** 2020-07-18

**Authors:** Elvira Lange, Inger Gjertsson, Kaisa Mannerkorpi

**Affiliations:** 1 Department of Health and Rehabilitation, Unit of Physiotherapy, Institute of Neuroscience and Physiology, The Sahlgrenska Academy, University of Gothenburg, Box 455, 405 30 Göteborg, Sweden; 2 University of Gothenburg Center for Person-centred Care, The Sahlgrenska Academy, University of Gothenburg, Gothenburg, Sweden; 3 Department of Rheumatology and Inflammation research, Institute of Medicine, The Sahlgrenska Academy, University of Gothenburg, Gothenburg, Sweden; 4 The Sahlgrenska University Hospital, Gothenburg, Sweden

**Keywords:** Rheumatoid arthritis, Physical activity, Physical activity

## Abstract

**Background:**

Physical activity and exercise are acknowledged as important parts in the management of rheumatoid arthritis (RA). However, long-term maintenance of exercise is known to be difficult. The aim of this study was to evaluate change in physical activity and physical fitness after four years in older adults with RA who had previously participated in exercise with person-centred guidance compared to controls.

**Method:**

A follow-up study was performed where older adults (> 65 years) who had participated in a randomized controlled trial where they were allocated to either exercise with person-centred guidance or home-based, light-intensity exercise (controls) were invited to one visit and assessed with performance-based test, blood-sampling and self-reported questionnaires. Forty-seven out of 70 older adults accepted participation, 24 from the exercise group and 23 from the control group. Comparisons of the result with baseline values were performed and explanatory factors for increase of physical activity were examined with logistic regression.

**Results:**

The result show that there was no significant difference in weekly hours of physical activity when groups where compared. However, participants in the exercise group rated significantly increased weekly hours of physical activity after four years (*p* = 0.004) when compared to baseline. Higher levels of fatigue, BMI and physical activity, at baseline were negatively associated with increased physical activity after four years. There was no significant difference in change of physical fitness between the groups. Within group analysis showed that the control group reported increased pain (*p* = 0.035), fatigue (*p* = 0.023) increased number of tender joints (*p* = 0.028) higher disease activity (*p* = 0.007) and worsening of global health (*p* = 0.004) when compared to baseline while the exercise group remained at the same level as at baseline.

**Conclusion:**

These results indicate that introducing moderate- to high intensity exercise with person-centred guidance might favor increased physical activity after four years in older adults with RA. Previous partaking in moderate- to high intensity exercise might also be protective against increased disease activity, pain and fatigue over time.

## Background

Physical activity, defined as all body movement resulting in increased energy expenditure, is well documented as health promoting and disease preventing, not least among older adults [1] and for persons with chronic diseases like rheumatoid arthritis (RA) [2]. Physical activity needs to be performed regularly and maintained over time to maintain health benefits and the World Health Organization (WHO) recommendations for health-enhancing physical activity comprise 150 min of moderate intensity aerobic exercise each week together with muscle-strengthening activities two times a week [1]. These recommendations align with the 2018 EULAR recommendations for physical activity in people with inflammatory arthritis and osteoarthritis, where it is advocated that physical activity should be part of standard care (ref). In patients with RA these recommendations are important not only for general health benefits but for improvements of disease activity, activities and participation [3].

RA is an inflammatory joint disease with pain, fatigue and disability as common symptoms [4]. Persons with RA present a lower physical capacity and less physical activity than healthy persons in the same age; this is particularly pronounced in older adults with RA [5]. Our research group has shown that older adults (> 65 years) with RA experiences moderate- to high intensity exercise with person-centred guidance as manageable [6] and that they gained good effect on physical fitness and rated their health as much improved [7]. Exercise with person-centred guidance also seemed to prepare the participants for independent exercise [6] and 7 months after completion of intervention the participants were still active to a large extent when compared to previous studies among adults with RA reporting maintenance of exercise after an intervention [8, 9].

Older adults have shown difficulties with long-term maintenance of exercise [10]. This is also a known problem for persons with RA who need to manage barriers for exercise, both disease-specific and general [11]. Three years after a successful exercise intervention for persons with RA less than 65 years, none of the participants had maintained exercise with a high intensity [12]. Current research is still not sufficient to determine which methods are effective in promoting maintenance of physical activity and exercise over time [13, 14].

The aim of this study was to evaluate change in level of physical activity and physical fitness after 4 years in participants who had previously participated in moderate- to high-intensity exercise with person-centred guidance compared to controls. The aim was also to study which factors that had influenced physical activity after 4 years.

## Methods

### Participants

**Fig. 1:**
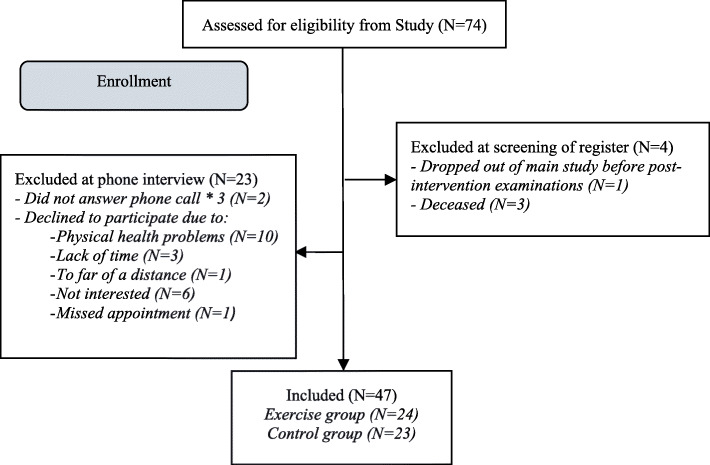
Flowchart of the inclusion of study participants

Participants was recruited among the 74 participants previously included in a randomized controlled intervention study [6] performed in 2015–2016 in Gothenburg and Skövde in the Region Västra Götaland, Sweden. All, now living, participants who participated in post-intervention assessments (*n* = 70) were invited by mail to participate (Fig. 1). In the intervention study participants were recruited from the Swedish Rheumatology Quality Register based on the inclusion criteria: RA according to the American College of Rheumatology 1987/European League Against Rheumatism 2010 criteria, aged ≥65 years, disease duration > 2 years, and low-to-moderate Disease Activity Score in 28 joints (DAS28 < 5.1). The exclusion criteria were comorbidities such as unstable ischemic heart disease or arrhythmia that might preclude moderate intensity exercise, joint surgery within 6 months prior to inclusion, ongoing exercise of moderate-to-high intensity ≥ 2 times/week, inability to understand or speak Swedish, and inability to participate in physical testing that involved walking or bicycling.

A flowchart of the enrollment is presented in Fig. 1.

In the intervention study the subjects were randomised to either moderate- to high-intensity exercise with person-centred guidance by a physiotherapist for 20 weeks or to the control intervention consisting of low-intensity home-exercise [7]. The intervention was performed 2–3 times each week, with aerobic exercise of approximately 70–89% of max heart rate for 9*3 min, and five resistance exercises of approximately 70–80% of 1 repetition max. Both groups were encouraged to perform health enhancing physical activity according to the WHO recommendations [1]. At two occasions during 7 months after the intervention the exercise group were offered light support over telephone. The contact was then ended and the natural course was done until the current study.

The present 4-year follow up study complied with the Declaration of Helsinki and was approved by the Swedish Ethical Review Authority (790–14 (2019–01406)). Informed, written consent was obtained from the patients before the examinations.

### Data collection

The participants were assessed at one occasion where performance-based tests, blood sampling and self-reported questionnaires where used together with questions on current and previous health status. For background variables the Health Assessment Questionnaire – Disability Index (HAQ-DI), was used to assess general disability [15]. Global health, general pain and general fatigue was reported on a 100 mm Visual Analogue scale (VAS) with a higher number indicating worse health, more pain and more fatigue. A disease activity score (DAS28) was registered based on erythrocyte sedimentation rate (ESR) and a 28 joint physical examination [16]. Body weight and length was collected to calculate Body Mass Index (BMI) as weight (kg) / [height (m)]^2^.

### Outcomes

For assessment of self-rated amount of physical activity, *Leisure Time Physical Activity Inventory* (LTPAI) was used. Hours of light, moderate, and vigorous leisure-time physical activities during the last week were reported and calculated to a total score [17]. The total score of LTPAI was presented together with results of the three levels separately. LTPAI was also used as dependent variable in a regression analysis.

*Modified Exercise Stage Assessment Instrument* (ESAI) was used to assess maintained, health-enhancing physical activity [9, 18]. It is a two-question questionnaire where health-enhancing physical activity is defined in accordance with the recommendations from WHO and answered through five response options based on the stages of changes described in the transtheoretical model [19]. The first question defines aerobic physical activity for ≥30 min in ≥5 days/week and the second question defines biweekly resistance exercise. The result of both questions combined is presented as health-enhancing physical activity and the questions are also reported separately.

For assessment of physical fitness, a selection of performance-based tests used in the intervention study was performed. *Timed Up and Go* (TUG) test were used to assess dynamic balance by asking the participants to rise from an armchair; walk a distance of 3 m as quickly as possible but still safely; walk back; and sit down [20]. The total time was recorded. The test has shown good reliability in a Swedish population of patients with RA and is sensitive to change [21, 22]. The *Sit To Stand* (STS) test was used to assess leg muscle strength [23] by recording the number of complete rises from sitting on a chair performed in 60 s. The test is validated and has been shown to correlate to muscle strength in the lower extremities in a Swedish middle-aged to aged population [24, 25]. A *bicycle endurance test* was performed [26] on an electronically braked cycle ergometer (M*onark Ergometer* 839 E; Monark Exercise AB, Vansbro, Sweden). After a 2-min warm-up period at 50 W, the participants cycled at the same constant power as at baseline of the intervention study [7], and when the level of exertion was rated as “Very hard” on the Borg rating of perceived exertion scale [27] the total time was registered.

### Statistics

Statistical analyses were made with the Statistical Package Software for the Social Sciences version 24.0 (IBM Corp., Armonk, NY, USA). Between groups comparisons were performed with the Mann-Whitney U test and paired with-in group comparisons were made with Wilcoxon signed rank test. Chi-square was used for comparison of proportions.

Explanatory factors were analysed with multivariate logistic regression. In the model the variable LTPAI was dichotomised as lower/constant (n = 15) or higher (n = 32) than baseline and used as dependent variable. For selection of independent variables binary univariable logistic regressions were performed with baseline data of variables listed in Tables 1 and 2 as independent variables. In the final model the significant variables from the univariable analysis were used. As independent variable VAS fatigue and BMI at baseline of the intervention study was used together with physical activity level (LTPAI) from the same time point.

**Table 1:** Background data of the study population 4 years after inclusion. Changes from baseline to 4 years are presented

	(*n* = 24) Exercise group	(*n* = 23) Control group	Between group	Δ 4 years-baseline	Within group	Δ 4 years-baseline	Within group
	n (%)	n (%)	*p*	Exercise group	*p*	Control group	*p*
Sex /female)	18 (75%)	18 (78.3%)					
RF	17 (71%)	16 (70%)	0.924				
CCP	20 (83%)	12 (52%)	0.130				
Erosiv	13 (54%)	13 (57%)	0.980				
	*Mean (SD)*	*Mean (SD)*					
	*Median (min;max)*	*Median (min;max)*					
Age (years)	73.5 (2.71)	74.0 (2.11)	0.323				
	73 (70–79)	74 (70–78)					
Disease	18.7 (10.33)	21.9 (10.7)	0.112				
duration	17.5 (6–49)	24 (7–53)					
DAS28	2.62 (0.76)	3.17 (0.87)	**0.043**	0.29 (0.98)	0.058	0.72 (1.05)	**0.007**
	2.53 (1.03;4.36)	2.92 (1.30;4.53)		0.31 (−1.96;2.01)		0.58 (−1.11;2.32)	
Tender joints	1 (1.7)	2 (2.2)	0.065	−0.4 (2.4)	0.474	1 (2.2)	**0.028**
	0 (0;6)	1 (0;8)		0(−7;5)		1 (−3;7)	
Swollen joints	0.3 (0.8)	0.7 (1.3)	0.327	−0.2 (1.4)	0.558	−0.1 (1.9)	0.811
	0 (0;3)	0 (0;5)		0 (−5;3)		0 (−6;4)	
ESR	18.8 (11.8)	17.7 (10.1)	0.918	6.9 (7.9)	**0.001**	5.5 (5.5)	**< 0.001**
	16.5 (5;53)	16 (3;38)		4.5 (−11;25)		6 (− 2;21)	
CRP	2.8 (2.0)	3.2 (3.1)	0.699	−1.0 (2.9)	0.124	−1.3 (5.3)	0.465
	2.5 (1;10)	2 (1;11)		0 (−11;3)		0 (−22;5)	
HAQ-DI	0.47 (0.51)	0.63 (0.54)	0.250	−0.02 (0.25)	0.374	−0.01 (0.39)	0.979
	0.31 (0;1.63)	0.5 (0;2.13)		0 (−0.5;0.75)		0 (−0.75;0.63)	
VAS,	21.8 (18.3)	39.6 (24.1)	**0.008**	0 (12.5)	0.658	13.7 (20.6)	**0.004**
Global Health	20.5 (1;69)	34 (2;98)		3 (−29;20)		11 (−17;65)	
VAS Pain,	23.5 (21.4)	35.8 (25.5)	0.081	3.2 (19.1)	0.539	14 (27)	**0.035**
current (mm)	19 (0–69)	28 (1;94)		1.5 (−32;62)		9 (−32;80)	
VAS Fatigue	32 (21.7)	40.7 (27.4)	0.229	5.7 (16.4)	0.124	11.7 (22.8)	**0.023**
	33.5 (0;68)	48 (0;86)		6 (−22;55)		6 (−37;61)	
Body mass	25.7 (5.1)	26.7 (4.0)	0.482	0.65 (3.7)	0.287	−0.93 (4.17)	0.648
index	26.0 (17.7–40.0)	26.4 (19.2;36.9)		0.30 (−7.72; 14.37)		0.003 (−18.7; 2.8)	

*RF Rheumatoid factor, CCP Cyclic Citrullinated Peptide, Disease duration years, DAS28 Disease Activity Scale 28, ESR Erythrocyte sedimentation rate, CRP C-reactive protein, HAQ-DI Health Assessment Questionnaire - Disability index, VAS Visual Analogue Scale*

*Significant p-values are shown in bold*

**Table 2:** Change of physical activity and fitness over four years

	Exercise group (n = 24)				Control group (n = 23)				
	Baseline	4 years	Δ 4 years-baseline	Within *p*	Baseline	4 years	Δ 4 years-baseline	Within *p*	Between groups, *p*
LTPAI, total	6.3 (6.1)	9.9 (7)	3.7 (7.1)	**0.004**	7 (4.4)	8.1 (4.5)	1.1 (6.1)	0.241	0.245
	4 (0;28)	8 (2;28)	3 (−15;27)		6 (0;19)	7 (2;20)	2 (−10;12)		
TUG, sec	7.6 (1.6)	8.2 (3.8)	0.6 (2.9)	0.758	8.2 (1.9)	8.1 (2.4)	−0.1 (1.8)	0.484	0.809
	7.1 (5;10.5)	7.2 (5;21,4)	−0.2 (−2.1;11.4)		7.6 (5.5;13)	7.3 (5.4;12.5)	−0.25 (−4,5;3.1)		
STS, n	22.5 (4.1)	22.7 (8.8)	0.5 (6.9)	0.229	21.2 (5.2)	21.7 (7)	0.1 (6)	0.675	0.750
	23 (15;30)	23 (0;42)	2 (−21;13)		21 (9;30)	23.5 (8,32)	0.5 (−15;9)		
Endurance,	11.3 (6.2)	6.3 (2.3)	−4.9 (6.8)	**0.002**	8.6 (4.3)	7.2 (6.1)	−1.4 (6.6)	0.056	0.191
min	9.5 (4;25)	6 (3;12)	−2 (−20;6)		6 (4;20)	5 (3;32)	−1.5(−15;21)		

*Values are presented as mean (SD) and median (min;max). Significant p-values are shown i bold. LTPAI Leisure time physical activity Index, TUG Timed up and go, STS Sit to stands test. Missing Δ values: Exercise group: TUG (n = 1), STS (n = 2), Endurance (n = 2). Controll group: STS (n = 1), Endurance (n = 1)*

## Results

Forty-seven older adults (67% of the 70 invited persons) accepted participation in this 4-year follow up. The mean length from inclusion in the intervention study was 3 years and 10 months (range: 3 years, 5 months to 4 years, 3 months). The included participants did not differ from those who declined in comparison of background variables at baseline of the intervention study. The primary reason for not participating was health problems (n = 10), for example lung problems or knee pain (Fig. 1). Three of the participants did not participate in all performance based test due to pain or symptoms of heart disease.

Background variables at 4-year follow up are presented in Table 1 together with a presentation of change over the last 4 years. The participants on the exercise group had a significantly lower DAS28 (p = 0.029) and VAS Global Health (p = 0.008) compared to the control group at 4-year follow up. The variable ESR was significantly increased over 4 years in both groups, when compared to baseline (Table 1). Disease activity assessed by DAS28 (p = 0.004), tender joints (p = 0.028), VAS pain (p = 0.035) and VAS fatigue (p = 0.023) was significantly increased and Global Health assessed with VAS was significantly worsened (p = 0.004) with-in the control group but not in the exercise group after 4 years. Four participants in the exercise group and five in the control group reported considerable changes of rheumatic medication between the intervention study and the 4-year follow up. The included participants where compared concerning comorbidities stated at baseline and self-reported sever medical events during the last 4 years. The groups where considered equal in distribution, with 14 participants in each group suffering from concomitant diseases. From a total of 24 participants in the exercise group: cardiovascular disease 2, hypothyroidism 4, diabetes mellitus 1, pulmonary disease 2, previous cancers 8, and other diseases 5, and from a total of 23 patients in the control group: cardiovascular disease 2, hypothyroidism 5, diabetes mellitus 2, pulmonary disease 2, previous cancers 5, and other diseases 5). Also, 4 persons in the exercise group and 5 persons in the control group had a joint prosthesis.

### Physical activity level

There was no significant difference between groups when change of self-rated total amount of physical activity after 4 years was compared between groups. When the self-rated total amount of physical activity was compared to baseline there was a significant (p = 0.004) increase of physical activity within the exercise group but not in the control group (Table 2). When LTPAI was divided in different levels of activity the light and moderate activity amount was significantly increased within the exercise group when compared to baseline of the interventions study (p < 0.05). There were no significant changes in vigorous activity and there were no significant differences when change was compared between groups (Table 2*).*

### ESAI

In the exercise group, 33.3% reported current health enhancing physical activity, the corresponding number in the control group was 26.1%. Maintained health enhancing physical activity was reported by 25% in the exercise group and 17.4% in the control group. There were no significant differences between groups (p = 0.560).

**Fig. 2:**
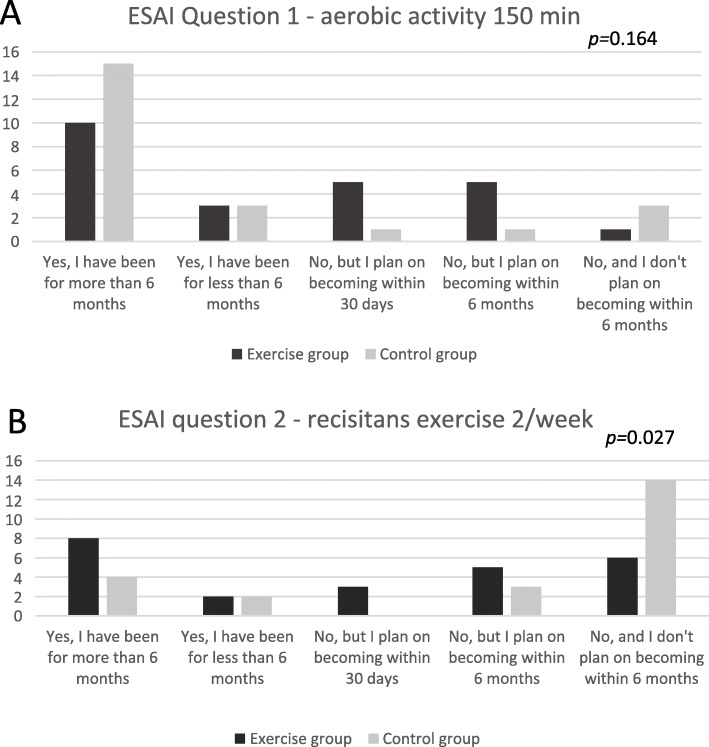
Response of the ESAI question 1(a) and 2 (b)

A majority of participants (25/47) stated that they had maintained 150 weekly minutes of aerobic physical activity for the last 6 months (Fig. 2a). There was no significant difference between groups (p = 0.164). Only eight participants in the exercise group and four in the control group stated that they had maintained resistance exercise twice a week for 6 months (Fig. 2b). Notably there were 14 participants of the control group stating that they did not plan to start with resistance exercise in 6 months while only six participants of the exercise group stated the same (p = 0.027).

### Physical fitness

There were no significant differences in change of the performance based tests between the groups *(*Table 2). No significant differences within either of the groups were found in TUG and STS between 4 years and baseline (0 years). The bicycle endurance test was significantly decreased from baseline (p = 0.002) within the exercise group.

### Association with increased hours of physical activity after 4 years

Higher level of fatigue *(VAS fatigue)*, BMI and physical activity *(baseline LTPAI)*, at baseline was negatively associated with increased physical activity after 4 years *(*Table 3*).*

**Table 3:** Result of logistic regression of association with LTPAI after four years

	Regression coefficient (B)	p-value	Odds ratio	95% C.I.for Odds ratio	
				Lower	Upper
LTPAI_0	−0.206	**0.012**	0.814	0.692	0.956
VAS_Fatigue_0	−0.046	**0.018**	0.955	0.920	0.992
BMI_0	−0.234	**0.022**	0.792	0.648	0.967
Constant	9.935	**0.002**	20,635.923		

*C.I. confidence interval. Significant p-values are shown in bold*

For every 1-unit increase in BMI at baseline participants were 21% less likely to increase their rated physical activity after 4 years (OR = 0.79, p = 0.022), while for every additional hour of physical activity at baseline the participants were 19% less likely to increase their rated physical activity after 4 years (OR = 0.81, p = 0.012). For every 1 mm higher rating of VAS fatigue at baseline, the participants were 4% less likely to increase their physical activity (OR = 0.96, p = 0.018).

The model explained 51.4% (Nagelkerke R squared) of the variance in LTPAI change. The model was statistically significant (p < 0.001), indicating that the model was able to differentiate respondents who rated increased LTPAI from those who did not and the model as a whole correctly classified 85.1% of the cases.

## Discussion

This study shows that higher levels of fatigue, BMI and physical activity at baseline were negatively associated with increased physical activity after 4 years. There was a significant difference between groups in rated global health, and the global health of the control group was significantly worsened compared to baseline. The DAS28, together with pain, fatigue and tender joints, was increased within the control group but not in the exercise group indicating that increased physical activity amount seems to have positive effects on the tender joint-component of disease activity in preventing a decline over time. However, the ESR increased in both groups, which is to be expected with increased age [28]. Exercise is known to possibly slow down the aging of the immune system and diminish the effects of “inflam-aging” [29–31] but neither in this 4 year follow-up study nor directly after the intervention were any changes of inflammatory markers found [32].

Of all participants 21.3% reported maintained health-enhancing physical activity (including both aerobic and resistance training) for the last 6 months before inclusion in the present study. That could be considered high when compared to a large Swedish cohort with RA aged 18–75 years of whom 11% maintained health enhancing physical activity [5]. Partaking in an exercise intervention could increase health enhancing physical activity [33]. It is well known that a decline in PE is expected over time [9]. Therefore, it is not surprising that the participants of this study did not maintain the high intensity of physical activity initiated in the exercise intervention despite still reporting health-enhancing physical activity. Such decline over 4 years could be related to different barriers for physical activity appearing over time. Older adults with RA describe impaired physical health as an important barrier for exercise [6, 34, 35]. The risk of encountering impaired physical health is considerable in older age as age is a risk factor for several diseases [36] and the prevalence of frailty increases with age [37]. The rheumatic disease itself is also a risk factor for impaired physical health due to flares [38] and increased risk for cardiovascular diseases [39] and other extra-articular complications [40]. None or short lapses in exercise are shown to predict maintenance of exercise so prevention of lapses could be important to promote maintenance [41]. Since health problems possibly cause lapses and relapses of exercise, continuous support for exercise might be needed to manage the challenge of declining physical health. With regular contact with health care in higher age, the physician is acknowledged as a key person to promote physical activity [35] and the physical activity level of the rheumatologist has shown to affect the level of promotion [42]. Professional support from a physiotherapist could likewise be important since physiotherapy aims to maintain and restore movement throughout the lifespan and could assist with enabling adjustments of exercise based on current health status and physical function [43]. The performed intervention of the intervention study was extensive and lead to adoption of exercise among most participants, but for long-lasting maintenance of exercise even more support might have been necessary. However, the light support offered during the first 7 months after the intervention was considered adequate [6] and the maintenance rate was high [7]. Through a person-centred adoption of exercise maintenance interventions and support an effective design could be reached considering both resources and results since person-centred care has been found to combine both positive outcomes and cost-effectiveness in other domains [44, 45].

Unlike the exercise group a majority of the participants in the control group did not consider starting with resistance exercise. This may be related to a view on aging [46] where resistance exercise is not thought of as suitable for older people and it could also be related to a lack of self-efficacy for resistance exercise. The exercise intervention was designed based on principles of increasing self-efficacy [47] using a person-centred approach and a slow, low-level introduction and continuous adjustments [7] supporting goal accomplishments and the experience of mastery [47]. The participants of the exercise group have expressed that they developed their thoughts about exercise and conquered the gym as a new arena during the intervention [6] which probably was a reason for the exercise group viewing themselves as possibly taking on resistance exercise in aging. Positive physical activity intentions and a more positive view on aging have been shown to be predictors of increased physical activity in people with RA [46, 48].

Previous studies show that an age-related decline in the measurements of physical fitness could have been expected [49–51] since muscle strength and power decline with age [52]. Within none of the groups no difference from baseline was found in TUG and STS indicating that both interventions might have slowed down the typical age-related decline of physical fitness. Even light-intensity physical activity is suggested to prevent physical decline in people with arthritis [53]. To increase fitness there is however a need for maintenance of structured physical activity [54]. The declining result of the endurance test within the exercise group might seem to contradict the finding of increased levels of physical activity within the exercise group. Despite increased amount of physical activity, endurance did not remain. The results of LTPAI might, however, capture aspects of physical activity that do not affect endurance as measured in the test, for example lightintensity physical activity and resistance exercise. Assessment of self-reported physical activity is known to be difficult [55] but could still be purposive [56] and the test-retest reliability of LTPAI has been found to be satisfactory [17].

The regression model found three significant variables predicting increase of physical activity rated on LTPAI over 4 years. Higher BMI at baseline was found to reduce the chance of increasing LTPAI with 21% for every additional step of BMI. Previous studies have found high BMI to negatively predict physical activity behaviour among adults [57, 58] and both direct and indirect effects have been suggested. A higher BMI could both add physical strains to performing exercise and be associated with social barriers [58].

The same model also indicated that a higher-rated fatigue at baseline decreases the chance of having increased level of physical activity after 4 years. In people with RA fatigue, this is, together with pain, the most common disease specific barrier to physical activity and exercise [11, 59]. Despite a personal experience of positive effects of adoption of physical activity on fatigue, ongoing hard work is required to overcome fatigue to be able to be physically active [60]. When fatigue is a long-lasting barrier for exercise, the mental load of continuously overcoming it might be a reason for fatigue negatively affecting physical activity level over time. For every rated mm (0–100) on VAS fatigue the chance of increasing physical activity over 4 years only decreased by 4%, but with clinically relevant fatigue being defined as VAS > 20 [61], a participant presenting a clinically relevant fatigue at baseline could be considered to be at 56% less chance of increasing LTPAI over 4 years. However, this cohort only showed limited fatigue at baseline and the intervention was found to reduce fatigue [32].

With change of LTPAI being the dependent variable in the model the baseline value of LTPAI was controlled for. In this study a higher amount of physical activity at baseline decreased the chance of having increased LTPAI after 4 years. Previous studies have found that past history of exercise or vigorous exercise at baseline could predict maintained exercise [42, 62], and previous exercise behavior has been found to correlate with physical activity level [63, 64]. In this study, baseline LTPAI only reported current physical activity and did not report previous exercise behavior and experiences. The results indicate that older adults with RA could probably experience an upper limit of weekly time (median baseline LTPAI = 6 h) that is reasonable to devote to exercise so that the potential of increasing exercise diminishes with an already high activity level. Lack of time and competing activities are known barriers for exercise [6, 11].

The strengths of the study were that the follow-up was performed after several years and that it contained on-site testing of performance and blood sampling. A limitation of the study was the small sample size and that 33% of participants in the intervention study was lost to this follow-up. The results should be interpreted with caution. However, the included participants did not differ in baseline background variables from those not participating.

## Conclusion

Older adults with RA, who previously had been partaking in an exercise intervention with person-centred guidance, rated significantly increased physical activity after 4 years when compared to baseline but the difference between groups was not significant indicating a need for long-lasting support for maintenance of exercise. Higher BMI, fatigue and amount of physical activity at baseline negatively predicted physical activity after 4 years.

Physical fitness was preserved at the same level in both groups after 4 years. An increase of disease activity, pain and fatigue among participants of the control group might indicate that introducing moderate- to high intensity exercise with person-centred guidance might be protective against such increase over time.

## Data Availability

Data will not be made available before all publishing from the study is completed.
